# The neuropeptide genes *SST*, *TAC1*, *HCRT*, *NPY*, and *GAL* are powerful epigenetic biomarkers in head and neck cancer: a site-specific analysis

**DOI:** 10.1186/s13148-018-0485-0

**Published:** 2018-04-11

**Authors:** Kiyoshi Misawa, Masato Mima, Atsushi Imai, Daiki Mochizuki, Yuki Misawa, Shiori Endo, Ryuji Ishikawa, Takeharu Kanazawa, Hiroyuki Mineta

**Affiliations:** 1 Department of Otolaryngology/Head and Neck Surgery, Hamamatsu University School of Medicine, 1-20-1 Handayama, Hamamatsu, Shizuoka 431-3192 Japan; 2 Department of Otolaryngology/Head and Neck Surgery, Jichi Medical University, Tochigi, Japan

**Keywords:** *SST*, *TAC1*, *HCRT*, *NPY*, *GAL*, Head and neck cancer, Epigenetic markers, Site-specific analysis

## Abstract

**Background:**

Staging and pathological grading systems are convenient but imperfect predictors of recurrence in head and neck squamous cell carcinoma (HNSCC). Identifying biomarkers for HNSCC that will progress and cause death is a critical research area, particularly if the biomarker can be linked to selection of patients. Therefore, to identify potential alternative prognostic markers, we investigated the methylation status of five neuropeptide gene promoters. The promoter methylation status was determined by quantitative methylation-specific PCR in 230 cases of HNSCC; 58 hypopharynx, 45 larynx, 56 oropharynx, and 71 oral cavity tumor samples were studied.

**Results:**

The somatostatin (*SST*), tachykinin precursor 1 (*TAC1*), hypocretin neuropeptide precursor (*HCRT*), neuropeptide Y (*NPY*), and galanin (*GAL*) promoters were methylated in 84.3, 63.5, 32.6, 28.3, and 20.0%, respectively, of the samples. The mean number of methylated genes per sample was 2.29 (range, 0–5). Disease-free survival was lower in patients with 3–5 methylated genes than in those with 0–2 methylated genes (log-rank test, *P* = 0.007). In multivariate Cox proportional hazards analysis, *TAC1* and *GAL* promoter methylation independently predicted recurrence (odds ratios 1.620, 95% confidence interval [CI] 1.018–2.578, *P* = 0.042, and odds ratios 1.692, 95% CI 1.063–2.694, *P* = 0.027, respectively). In patients with oral cancer, *TAC1* methylation showed the best correlation with poor survival (odds ratio 4.427, 95% CI 1.634–12.00, *P* = 0.003). Similar findings were observed for *HCRT* and *GAL* in patients with laryngeal cancer and oropharyngeal cancer, respectively.

**Conclusion:**

In this study, we demonstrated the methylation status of the neuropeptide-encoding genes *SST*, *TAC1*, *HCRT*, *NPY*, and *GAL* and its relationship with recurrence and survival in HNSCC. These methylation changes may serve as potential molecular markers for defining the risk and prognosis of HNSCC.

**Electronic supplementary material:**

The online version of this article (10.1186/s13148-018-0485-0) contains supplementary material, which is available to authorized users.

## Background

Neuropeptides and their receptors are important messenger molecules that carry information between neurons; they can act as neurohormones, neurotransmitters, and neuromodulators and maintain physiological homeostasis [1, 2]. Neuroendocrine peptides play essential roles in the regulation of gastrointestinal endocrine and exocrine secretion, motility, and mucosal immunity. Moreover, some neuroendocrine peptides such as *gastrin*, *vasoactive intestinal peptide*, and *neurotensin* have been implicated in the modulation of human tumorigenesis [3, 4]. Most neuropeptides exert their effect through G protein-coupled receptors (GPCRs), with some exceptions [4]. GPCRs belong to a superfamily of cell surface signaling proteins that play a pivotal role in many physiological functions and multiple diseases [5]. Recent data have indicated that many GPCRs and their ligands are involved in cancer initiation and progression, including processes such as aberrant cell proliferation, invasion, metastasis, migration, adhesion, and angiogenesis [6]. The expression and secretion of neuropeptides by tumors has attracted increasing interest, as these peptides have been found to influence tumor proliferation and to be correlated with the appearance of characteristic clinical symptoms [7].

Staging and pathological grading systems are convenient but imperfect predictors of recurrence in head and neck squamous cell carcinoma (HNSCC). In HNSCC, methylation of gene promoters is a common mechanism of transcriptional silencing [8]. We recently defined the methylation profiles of the neuropeptide genes somatostatin (*SST*), tachykinin precursor 1 (*TAC1*), and galanin (*GAL*) and their cognate receptor gene members in HNSCC tumors [9–11]; however, further studies were required because of the small sample size and the lack of discrimination between the sites of origin of primary tumors. Nevertheless, it is evident that the neuropeptide system functions as a major mechanism in HNSCC carcinogenesis.

The current findings provide novel direct epigenetic evidence for the involvement of *SST*, *TAC1*, hypocretin neuropeptide precursor (*HCRT*), neuropeptide Y (*NPY*), and *GAL* in the process of tumor suppression in humans. The association between the methylation status of the five genes and clinicopathological characteristics (e.g., tumor location and lymph node metastasis) was also assessed. To our knowledge, this study is the first to implicate neuropeptide gene methylation in the genesis of HNSCC.

## Methods

### Tumor samples

All clinical specimens were surgically obtained from primary HNSCCs (*n* = 230) at Hamamatsu University School of Medicine. The samples were obtained soon after diagnosis and were thus from untreated tumors. All patients provided written informed consent, and the study protocol was approved by the Institutional Review Board of the Hamamatsu University School of Medicine. Pertinent information including age, sex, smoking status, alcohol consumption, tumor site, size, lymph node status, and clinical stage was obtained from the patients’ medical records. The male to female ratio in the patient cohort was 193:37. The mean age was 65.5 years (range, 32–93 years). Primary tumors were in the hypopharynx (*n* = 58), larynx (*n* = 45), oropharynx (*n* = 56), or oral cavity (*n* = 71).

### Quantitative methylation-specific PCR (Q-MSP) analysis

Extraction and bisulfite conversion of genomic DNA from 230 primary HNSCC and 36 noncancerous mucosal samples were performed using the MethylEasy Xceed Rapid DNA Bisulfite Modification Kit (TaKaRa, Tokyo, Japan) per the manufacturer’s instructions [12]. The methylation levels of the CpG islands in the promoters of the *SST*, *TAC1*, *HCRT*, *NPY*, and *GAL* genes were determined via Q-MSP with the TaKaRa Thermal Cycler Dice Real Time System TP800 (TaKaRa); the primer sets are listed in Additional file 1: Table S1. Exon structure and CpG sites within expanded views of the promoter region relative to the transcription start site (TSS) are presented in Additional file 2: Figure S1. A standard curve was constructed by plotting known concentrations of serially diluted EpiScope Methylated HeLa gDNA (TaKaRa). The normalized methylation value (NMV) was determined as follows: NMV = (Target gene-S/Target gene-FM)/(ACTB-S/ACTB-FM), where Target gene-S and Target gene-FM represent the target gene methylation levels in the tumor sample and universal methylated DNA control, respectively, and ACTB-S and ACTB-FM represent the *ACTB* (which encodes β-actin) methylation levels in the sample and control, respectively. Analysis was performed using the software (version 1.03A) for the Thermal Cycler Dice Real Time System TP800 (TaKaRa), according to the manufacturer’s directions [13].

### Collection of publicly available data from The Cancer Genome Atlas (TCGA)

Aberrant DNA methylation data available in the TCGA (November 2017) were collected via the MethHC database (http://methhc.mbc.nctu.edu.tw/php/index.php) by using the Infinium HumanMethylation450 platform (Illumina, Inc., San Diego, CA, USA) and were expressed as *β* values. The *β* value is a number between 0 (not methylated) and 1 (completely methylated) that represents the ratio of methylated allele intensity and overall intensity [14].

### Data analysis and statistics

The Q-MSP results and patient characteristics (age of onset, sex, alcohol consumption, smoking status, tumor size, tumor stage, clinical stage, lymph node status, and recurrence) were compared by using the chi-squared test and Student’s *t* test. Receiver operating characteristic (ROC) curve analysis was performed using the NMVs for 36 HNSCC and 36 adjacent normal mucosal samples in the Stata/SE 13.0 system (Stata Corporation, TX, USA). The area under the ROC curve indicated the optimal sensitivity and specificity cutoff levels for distinguishing between the methylation levels in normal and HNSCC tissue, and the NMV thresholds were calculated for each target gene (Additional file 3: Figure S2). The cutoff values were used to determine the methylation frequencies of the target genes. The overall methylation rates in the individual samples were determined by calculating the methylation index (MI). MI was defined as the ratio of the number of methylated genes to the number of tested genes in each sample [13].

Disease-free survival (DFS) was measured from the date of the initial treatment to the date of diagnosis of locoregional recurrence or distant metastasis. The Kaplan-Meier test was used to calculate survival probability, and the log-rank test was used to compare survival rates. The prognostic value of methylation status was assessed by performing multivariate Cox proportional hazards analysis which was performed with adjustment for age (≥ 65 versus < 65 years), sex, smoking status, alcohol intake, and tumor stage (I and II versus III and IV). Differences with *P* < 0.05 were considered significant. All statistical analyses were performed using StatMate IV software (ATMS Co. Ltd., Tokyo, Japan).

## Results

### Methylation status of neuropeptide gene promoters

**Fig. 1:**
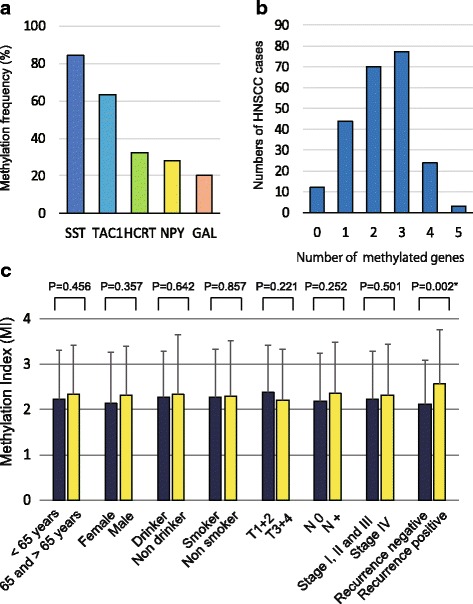
Methylation of the *SST*, *TAC1*, *HCRT*, *NPY*, and *GAL* gene promoters in 230 HNSCC samples. **a** Bar graph showing the methylation frequencies of the five genes. **b** Bar graph comparing the number of HNSCC cases to the number of methylated genes. **c** Double bar graph showing the methylation indices (MIs) according to the selected clinical parameters. The mean MI for each parameter was determined by using Student’s *t* test. Mean and standard deviation are also indicated, and statistical comparisons between groups are depicted. A probability of < 0.05 (*P < 0.05) was considered to represent a statistically significant difference

Q-MSP was used to assess the promoter methylation status of five genes encoding neuropeptide receptors in 230 primary HNSCC samples. The methylation rates for the five genes were as follows: *SST*, 84.3%; *TAC1*, 63.5%; *HCRT*, 32.6%; *NPY*, 28.3%; and *GAL*, 20.0% (Fig. 1a). At least one of these genes was methylated in most of the samples (218 of 230 samples, 94.8%). The mean number of methylated genes per sample was 2.29 (range, 0–5; Fig. 1b).

### Correlation between the methylation status of neuropeptide gene promoters and clinicopathological parameters

The associations between the methylation status of the target genes and the clinicopathological features of the patients are summarized in Table 1. The methylation of the *GAL* promoter was significantly correlated with recurrence events (*P* = 0.004). A trend toward higher recurrence rate was observed for patients with methylated *TAC1* (*P* = 0.051; Table 1). Methylation of *TAC1* was associated with smoking status (*P* = 0.007).

**Table 1:** Distribution of methylation status by selected epidemiologic and clinical characteristics

Characteristics					Age				Gender					Smoking status			
Gene	Methylation status		Overall(%)		< 65	> 65		*P* ^†^	Female		Male	*P* ^†^		Smoker	Non-smoker		*P* ^†^
SST	Yes		194(84.3)		81	113			29		165			146	48		
	No		36(15.7)		17	19		1	8		28	1		26	10		1
TAC1	Yes		146(63.5)		59	87			18		128			118	28		
	No		84(36.5)		39	45		1	19		65	0.061		54	30		0.007*
HCRT	Yes		75(32.6)		35	40			7		68			52	23		
	No		155(67.4)		63	92		1	30		125	0.057		120	35		1
NPY	Yes		65(28.3)		24	41			11		54			44	21		
	No		165(71.7)		74	91		0.302	26		139	1		128	37		1
GAL	Yes		46(20.0)		19	27			14		32			30	16		
	No		184(80.0)		79	105		0.869	23		161	1		143	42		1
Characteristics			Alcohol exposure			Tumor size			Lymph node status			Stage			Recurrence events		
Gene	Methylation status	Overall(%)	Drinker	Non-drinker	*P* ^†^	T1–2	T3–4	*P* ^†^	N0	N+	*P* ^†^	I, II, III	IV	*P* ^†^	Positive	Negative	*P* ^†^
SST	Yes	194(84.3)	140	54		95	99		80	114		86	108		78	116	
	No	36(15.7)	25	11	1	16	20	0.717	19	17	0.206	17	19	1	13	23	0.713
TAC1	Yes	146(63.5)	112	34		70	76		59	87		64	82		65	81	
	No	84(36.5)	53	31	1	41	43	1	40	44	1	39	45	1	26	58	0.051
HCRT	Yes	75(32.6)	55	20		39	36		28	47		30	45		37	38	
	No	155(67.4)	110	45	0.756	72	83	0.482	71	84	0.257	73	82	0.326	54	101	1
NPY	Yes	65(28.3)	44	21		37	28		32	33		30	35		26	39	
	No	165(71.7)	121	44	1	74	91	0.109	67	98	1	73	92	1	65	100	1
GAL	Yes	46(20.0)	25	21		23	23		18	28		20	26		27	19	
	No	184(80.0)	140	44	1	88	96	1	81	103	0.619	83	101	0.87	64	120	0.004*

*P < 0.05

†Chi-squared test

The mean differences in the MI according to the age of onset, sex, alcohol consumption, smoking habit, tumor size, lymph node status, clinical stage, and recurrence are illustrated in Fig. 1c. The MI was significantly higher in recurrence-positive cases than in recurrence-negative cases (*P* = 0.002; Fig. 1c).

### Site-specific analysis of the methylation status of neuropeptide gene promoters

**Fig. 2:**
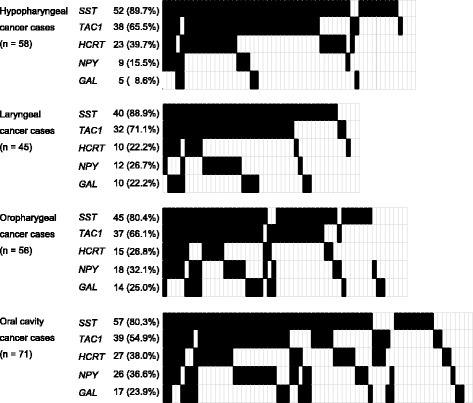
Comparison of methylation status of the promoters of five genes in patients with hypopharyngeal cancer, laryngeal cancer, oropharyngeal cancer, or oral cancer. Filled boxes indicate the presence of methylation, and open boxes indicate the absence of methylation

**Fig. 3:**
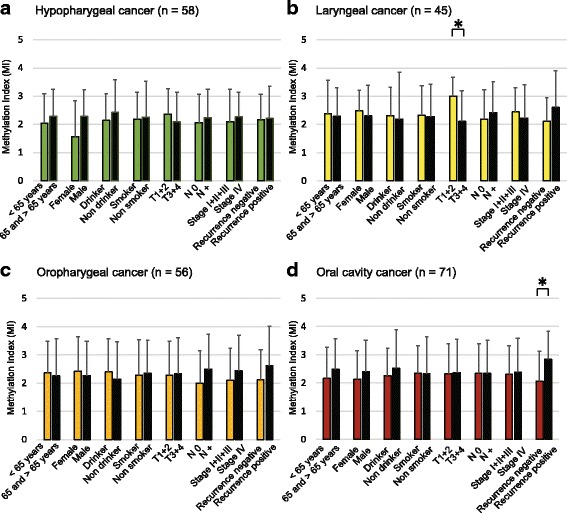
Association between MI and the selected clinical parameters. The mean MI for the various groups was compared using Student’s *t* test. Association between MI and selected epidemiologic and clinical characteristics: **a** hypopharyngeal cancer: no differences were noted with regard to any of the clinical characteristics; **b** laryngeal cancer: statistically significant differences were found for the associations between MI and tumor size; **c** oropharyngeal cancer: no differences were noted with regard to any of the clinical characteristics; and **d** oral cavity cancer: statistically significant differences were found for the associations between MI and recurrence events (positive versus negative). Mean and standard deviation are also indicated, and statistical comparisons between groups are depicted. A probability of < 0.05 (*P < 0.05) was considered to represent a statistically significant difference

Site-specific methylation frequencies of five genes for the hypopharynx (*n* = 58), larynx (*n* = 45), oropharynx (*n* = 56), and oral cavity (*n* = 71) are shown in Fig. 2. There was no significant association between clinicopathological characteristics and the MI in patients with hypopharyngeal cancer (Fig. 3a). Among laryngeal cancers, the MI was significantly higher in T1–2 than in T3–4 (*P* = 0.018; Fig. 3b). In patients with oropharyngeal cancer, no correlation was found between the MI and clinicopathological characteristics (Fig. 3c). We found that the MI was significantly higher in recurrence-positive cases than in recurrence-negative cases of oral cavity cancers (*P* = 0.004; Fig. 3d).

### Kaplan-Meier analysis

**Fig. 4:**
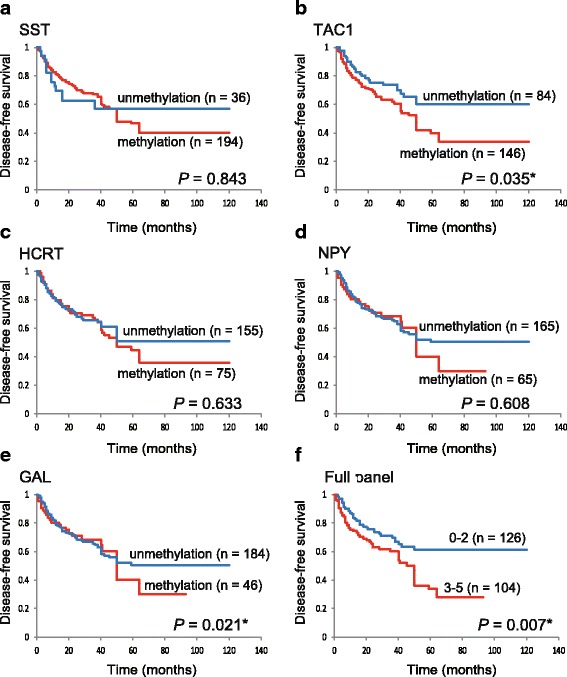
Kaplan-Meier survival curves for the 230 patients with HNSCC, according to the methylation status of the five target genes. Disease-free survival for **a** *SST*, **b** *TAC1*, **c** *HCRT*, **d** *NPY*, and **e** *GAL* in the case of methylated (red lines) and unmethylated (blue lines) genes. **f** Joint analysis of the five genes. Blue line: patients with 0–2 methylated genes; red line: patients with 3–5 methylated genes. A probability of < 0.05 (*P < 0.05) was considered to represent a statistically significant difference

The Kaplan-Meier survival curves for each of the five target genes are shown in Fig. 4. DFS time did not significantly differ between patients with methylated genes and those with unmethylated genes, with two notable exceptions: it was significantly shorter when *TAC1* was methylated (*P* = 0.035; Fig. 4b) and when *GAL* was methylated (*P* = 0.021; Fig. 4e). Based on log-rank tests, we detected an association between poor survival and the methylation phenotype defined as ≥ 3 methylated genes (*P* = 0.007; Additional file 4: Table S2). The DFS in patients with 3–5 methylated genes was lower than that in the group with 0–5 methylated genes (28.1 versus 61.3%, respectively; log-rank test, *P* = 0.007; Fig. 4f). Site-specific DFS time did not differ significantly in patients with methylated versus unmethylated genes, with two notable exceptions: it was significantly shorter when *SST* was unmethylated in laryngeal cancer (log-rank test, *P* = 0.001) and when *TAC1* was methylated in oral cavity cancer (log-rank test, *P* = 0.001) (Additional file 5: Figure S3, Additional file 6: Figure S4, Additional file 7: Figure S5, and Additional file 8: Figure S6).

### Prognostic value of the methylation status of neuropeptide gene promoters

The association between methylation and risk of recurrence was estimated via multivariate analysis by using a Cox proportional hazards model adjusted for age, sex, smoking status, alcohol consumption, and clinical stage. In patients whose *TAC1* promoter was methylated (146/230, 63.5%), the adjusted odds ratio (OR) for recurrence was 1.620 (95% confidence interval [CI] 1.018–2.578, *P* = 0.042). *GAL* methylation (46/230, 20.0%) showed a significant association with the OR for recurrence (OR = 1.692, 95% CI 1.063–2.694, *P* = 0.027; Table 2). Dense methylation of MI (3–5) had a significantly higher OR than low methylation of MI (0–2) for recurrence of 1.756 (95% CI 1.160–2.659, *P* = 0.008; Table 2).

**Table 2:** Methylation status of individual genes and associations with disease-free survival using Cox proportional hazards model in 230 patients

Gene	Methylation status	Overall(%)	Recurrence events		Adjusted RR (95% CI)^†^
			Positive (*N* = 91)	Negative (*N* = 139)	
SST	Yes	194(84.3)	78	116	
	No	36(15.7)	13	23	0.847 (0.470–1.526)
TAC1	Yes	146(63.5)	65	81	
	No	84(36.5)	26	58	1.620 (1.018–2.578)*
HCRT	Yes	75(32.6)	37	38	
	No	155(67.4)	54	101	1.235 (0.805–1.895)
NPY	Yes	65(28.3)	26	39	
	No	165(71.7)	65	100	1.126 (0.713–1.779)
GAL	Yes	46(20.0)	27	19	
	No	184(80.0)	64	120	1.692 (1.063–2.694)*
MI	3–5	104(45.2)	53	51	
	0–2	126(54.8)	38	88	1.756 (1.160–2.659)*

*P < 0.05

^†^Adjusted for age, gender, smoking status, alcohol exposure, and stage

**Fig. 5:**
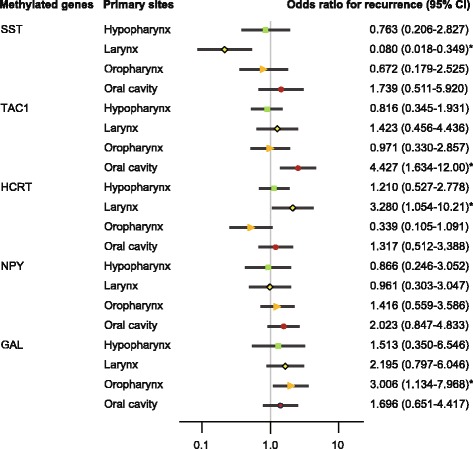
Risk of recurrence, based on gene methylation in tumors with different origins. Odds ratios for recurrence were determined using a Cox proportional hazards model adjusted for age (≥ 65 versus < 65 years), sex, smoking status, alcohol intake, and stage (I–II versus III–IV). CI: confidence interval

ORs for recurrence according to tumor origin were also determined for four sites in this study: the hypopharynx, larynx, oropharynx, and oral cavity. When the *SST* promoter was methylated in patients with laryngeal cancers, the OR was 0.080 (95% CI 0.018–0.349; *P* = 0.001). *TAC1* methylation in oral cancer was significantly associated with the OR for recurrence (OR = 4.427, 95% CI 1.634–12.00, *P* = 0.003). In patients who had laryngeal cancer and whose *HCRT* promoter was methylated, the adjusted OR for recurrence was 3.280 (95% CI 1.054–10.21; *P* = 0.040). Methylation of the *GAL* promoters was positively correlated with recurrence in patients with oropharyngeal cancers (OR = 3.006, 95% CI 1.134–7.968, *P* = 0.027; Fig. 5).

### External validation of results by using data from the TCGA database

The methylation status of the five neuropeptide gene promoters was determined in an additional 516 HNSCC samples and 50 normal samples (Additional file 9: Figure S7). The average *β* values for *SST*, *TAC1*, *NPY*, and *GAL* methylation were significantly higher in the HNSCC samples than in the normal samples (*P* < 0.05). Methylation of the *HCRT* and *GAL* promoters was not associated with patients with HNSCC or the normal control group. A Pearson correlation revealed significant correlations between mRNA expression and DNA methylation of *SST*, *TAC1*, and *NPY*, but not correlations of *HCRT* and *GAL* (Additional file 10: Figure S8). Recurrence events were significantly associated with *HCRT* methylation (*P* = 0.005) and *GAL* methylation (*P* = 0.031) in laryngeal cancer patients (Additional file 11: Table S3).

## Discussion

Identifying epigenetic modifications in *SST*, *TAC1*, *HCRT*, *NPY*, and *GAL* is important for understanding how tumors arise and whether they will recur. Using real-time PCR, we examined the methylation status of these genes, all of which encode neuropeptide genes, in 230 HNSCCs originating in the hypopharynx, larynx, oropharynx, or oral cavity. We found that aberrant methylation of the *TAC1* and *GAL* promoters was positively correlated with recurrence in patients with HNSCCs. To our knowledge, this study is the first to analyze whether HNSCC primary tumors originating from different anatomic sites exhibit similar DNA methylation changes or whether DNA methylation events are specific to the anatomic site.

*SST* and the somatostatin receptors have been identified as tumor suppressor genes that possess potent antitumor and antisecretory activities in several human cancers [15]. Hypermethylation of *SST* has been described in colon cancer [16], gastric cancer [17], esophageal cancer [18], and head and neck cancer [9]. *TAC1* encodes the neuropeptides substance P, neurokinin A, and neurokinin B, which act through three types of tachykinin receptors (*TACR1*, *TACR2*, and *TACR3*) [19]. Hypermethylation of *TAC1* is associated with poor prognosis in esophageal cancer, breast cancer, and colorectal cancer [20–22]. *HCRT* encodes a hypothalamic neuropeptide precursor protein that gives rise to two mature neuropeptides, orexin A and orexin B [23]. Orexins induce high levels of apoptosis, resulting in a massive reduction in cell growth in various cancer cell lines acting at *HCRTR1* or *HCRTR2* [24]. *NPY* activates five GPCRs, namely, *NPY1R*, *NPY2R*, *NPY4R*, *NPY5R*, and *NPY6R* [25]. It is one of the most abundantly distributed neurotransmitters and vasoconstrictors in the central and peripheral nervous systems. Hypermethylation of *NPY* has been described in several cancers, including renal cell carcinoma [26], breast cancer [27], and colorectal cancer [28]. *GAL* is involved in the regulation of many physiological functions, such as feeding, metabolism, and body weight control [29]. DNA methylation of *GAL* in the prefrontal cortex plays a role in major depressive disorder [30]. In HNSCC, recent data are conflicting, with significant *GAL* overexpression reported in tumor samples [31], whereas our previous study proposed that *GAL* promoter methylation and gene silencing is correlated with significantly lower DFS and growth suppression of HNSCC cells after forced gene expression [32].

Neuropeptides have become a focus area of research because of the distinct GPCR activation, potent and varied biological activities, and potential therapeutic targets [33]. A novel approach to treat insomnia, the most common sleep disorder, has been introduced recently with the approval of suvorexant, the first in a new class of orexin receptor (*HCRTR*) antagonists [34]. Newly developed neurokinin-1 receptor antagonists, especially aprepitant, have been recently analyzed for the prevention of postoperative nausea and vomiting [35]. Certain neuropeptides, which are natural compounds by origin, possess powerful antitumorigenic and tumor suppressor properties, thereby providing added benefits for future potential therapeutic strategies [36]. Hence, innovating to obtain more potent selective ligands (agonists) will be a focus area for specific tumor targeting in the future.

HNSCC is the most complex “organ site,” affecting different anatomic sites of the upper aerodigestive tract such as the oral cavity, larynx, and naso-, oro-, and hypopharynx [37]. Patients with HNSCC often present a long history of tobacco and alcohol use. Recently, human papillomavirus (HPV) infection has emerged as an additional risk factor [38]. Many other risk factors are also related to HNSCC; therefore, it is necessary to investigate the profiles for different anatomic sites [39, 40].

## Conclusion

The current study provides evidence that the methylation status of *SST*, *TAC1*, *HCRT*, *NPY*, and *GAL* is an independent prognostic factor for DFS in patients with HNSCC. Further analyses showed that the aberrant methylation of *SST* may be a potential marker for patients with laryngeal cancer that is at low risk of relapse. Moreover, to our knowledge, our study is the first to suggest that *TAC1*, *HCRT*, and *GAL* methylation is associated with worse DFS and that this may be a critical event in oral cancers, laryngeal cancers, and oropharyngeal cancers, respectively. This study involving human specimens and high-throughput profiling platforms may be susceptible to measurement bias from various sources. Our findings support the use of methylation markers in patient selection for adjuvant therapy after initial surgical treatment; however, our preliminary findings need to be validated in larger and more homogeneous HNSCC patient cohorts.

## Additional files

Additional file 1: **Table S1.** Real-time MSP primer list. (DOCX 20 kb)

Additional file 2: **Figure S1.** Schematic representation of methylation analysis of 0 on 5 genes by qMSP. Schematic representation of (a) *SST*, (b) *TAC1*, (c) *HCRT*, (d) *NPY*, and (e) *GAL* genes. Exon structures and CpG sites within expanded views of the promoter region relative to the transcription start site (TSS). Vertical lines, individual CpG sites; straight arrows, relative location of the primers used for qMSP; bent arrow, TSS; arrowhead, translation start site (ATG). (EPS 1793 kb)

Additional file 3: **Figure S2.** ROC curves for the methylation markers in head and neck carcinomas versus adjacent normal mucosal tissue. On the basis of the ROC curve analysis, the sensitivity, specificity, and cutoff level were determined to be 80.6%, 94.4%, and 0.046 for *SST* (a); 72.2%, 97.2%, and 0.08 for *TAC1* (b); 67.6%, 97.2%, and 0.099 for *HCRT* (c); 50.0%, 97.2%, and 0.041 for *NPY* (d); and 25.0%, 86.1%, and 0.100 for *GAL* (e). (EPS 1057 kb)

Additional file 4: **Table S2.** Results of log-rank tests for the effect of number of methylated genes on disease-free survival in 230 HNSCC cases. (DOCX 14 kb)

Additional file 5: **Figure S3.** Kaplan-Meier survival curves for the 58 patients with hypopharyngeal cancer, according to the methylation status of the five target genes. Disease-free survival for (a) *SST*, (b) *TAC1*, (c) *HCRT*, (d) *NPY*, and (e) *GAL* in the case of methylated (red lines) and unmethylated (blue lines) genes. (f) Joint analysis of the 5 genes. Blue line: patients with 0–2 methylated genes; red line: patients with 3–5 methylated genes. A probability of < 0.05 (*P < 0.05) was considered to represent a statistically significant difference. (EPS 1460 kb)

Additional file 6: **Figure S4.** Kaplan-Meier survival curves for the 45 patients with laryngeal cancer, according to the methylation status of the five target genes. Disease-free survival for (a) *SST*, (b) *TAC1*, (c) *HCRT*, (d) *NPY*, and (e) *GAL* in the case of methylated (red lines) and unmethylated (blue lines) genes. (f) Joint analysis of the 5 genes. Blue line: patients with 0–2 methylated genes; red line: patients with 3–5 methylated genes. A probability of < 0.05 (*P < 0.05) was considered to represent a statistically significant difference. (EPS 1471 kb)

Additional file 7: **Figure S5.** Kaplan-Meier survival curves for the 56 patients with oropharyngeal cancer, according to the methylation status of the five target genes. Disease-free survival for (a) *SST*, (b) *TAC1*, (c) *HCRT*, (d) *NPY*, and (e) *GAL* in the case of methylated (red lines) and unmethylated (blue lines) genes. (f) Joint analysis of the 5 genes. Blue line: patients with 0–2 methylated genes; red line: patients with 3–5 methylated genes. A probability of < 0.05 (*P < 0.05) was considered to represent a statistically significant difference. (EPS 1460 kb)

Additional file 8: **Figure S6.** Kaplan-Meier survival curves for the 71 patients with oral cavity cancer, according to the methylation status of the five target genes. Disease-free survival for (a) *SST*, (b) *TAC1*, (c) *HCRT*, (d) *NPY*, and (e) *GAL* in the case of methylated (red lines) and unmethylated (blue lines) genes. (f) Joint analysis of the 5 genes. Blue line: patients with 0–2 methylated genes; red line: patients with 3–5 methylated genes. A probability of < 0.05 (*P < 0.05) was considered to represent a statistically significant difference. (EPS 1465 kb)

Additional file 9: **Figure S7.** DNA methylation data from The Cancer Genome Atlas database. DNA methylation data for *SST*, *TAC1*, *HCRT*, *NPY*, and *GAL* were collected from the TCGA database of DNA methylation and gene expression in human cancer (http://methhc.mbc.nctu.edu.tw/php/index.php). *P < 0.05. (EPS 896 kb)

Additional file 10: **Figure S8.** Methylation and expression status of the five neuropeptide genes in HNSCCs in the TCGA database. Scatter plot analysis for (A) *SST*, (B) *TAC1*, (C) *HCRT*, (D) *NPY*, and (E) *GAL*. Spearman rank correlation coefficient (R2) and *P* values are shown. (EPS 2568 kb)

Additional file 11: **Table S3.** Distribution of methylation status by recurrence events in TCGA cohort. (DOCX 17 kb)

## Abbreviations

ACTB: β-Actin
CI: Confidence interval
DFS: Disease-free survival
GAL: Galanin
GPCR: G protein-coupled receptors
HCRT: Hypocretin neuropeptide precursor
HNSCC: Head and neck squamous cell cancer
HPV: Human papilloma virus
MI: Methylation index
NMV: Normalized methylation value
NPY: Neuropeptide Y
OR: Odds ratios
Q-MSP: Quantitative methylation-specific PCR
ROC: Receiver operating characteristic
SST: Somatostatin
TAC1: Tachykinin precursor 1
TACR: Tachykinin receptor
TCGA: The Cancer Genome Atlas
