# Supplementary material for: The neuropeptide genes SST, TAC1, HCRT, NPY, and GAL are powerful epigenetic biomarkers in head and neck cancer: a site-specific analysis
Source: Clin Epigenetics. 2018 Apr 11;10:52. doi: 10.1186/s13148-018-0485-0 (PMC5896056; doi:10.1186/s13148-018-0485-0)
Supplement: Supplementary file 1 — Table S1. Real-time MSP primer list. (DOCX 20 kb) [file 13148_2018_485_MOESM1_ESM.docx]

Additional file 1: Table S1. Real Time MSP Primer List

Gene

SST

TAC1

HCRT

NPY

GAL

ACTB

Forward/Reverse

F

R

F

R

F

R

F

R

F

R

F

R

Sequence

GGGGCGTTTTTTAGTTTGACGT

AACAACGATAACTCCGAACCTCG

GGCGGTTAATTAAATATTGAGCAGAAAGTCGC

AAATCCGAACGCGCTCTTTCG

TGATTATGGGTCGTCGCGTA

AACTATCCTCCGAACGCGAC

GTCGCGGCGAGGAAGTTTTA

ACTATACTATCGAACGAAACG

TGACGCGATTTCGGGCGGTT

TATCCGCCGCCCGATATAAC

TGGTGATGGAGGAGGTTTAGTAAGT

AACCAATAAAACCTACTCCTCCCTTAA

Length (bp)

106

123

103

100

82

133
