# Supplementary material for: The neuropeptide genes SST, TAC1, HCRT, NPY, and GAL are powerful epigenetic biomarkers in head and neck cancer: a site-specific analysis
Source: Clin Epigenetics. 2018 Apr 11;10:52. doi: 10.1186/s13148-018-0485-0 (PMC5896056; doi:10.1186/s13148-018-0485-0)
Supplement: Supplementary file 4 — Table S2. Results of log-rank tests for the effect of number of methylated genes on disease-free survival in 230 HNSCC cases. (DOCX 14 kb) [file 13148_2018_485_MOESM4_ESM.docx]

| Additional file 4: Table S2. Results of log-rank tests for effect of number of methylated genes on disease free survival in 230 HNSCC | | |
| --- | --- | --- |
| No. methylated genes | No. patients with profile | P |
| ≥0 | 230 |  |
| ≥1 | 218 | 0.198 |
| ≥2 | 173 | 0.309 |
| ≥3 | 104 | 0.007* |
| ≥4 | 26 | 0.087 |
| ≥5 | 3 | 0.063 |
