# Supplementary material for: The neuropeptide genes SST, TAC1, HCRT, NPY, and GAL are powerful epigenetic biomarkers in head and neck cancer: a site-specific analysis
Source: Clin Epigenetics. 2018 Apr 11;10:52. doi: 10.1186/s13148-018-0485-0 (PMC5896056; doi:10.1186/s13148-018-0485-0)
Supplement: Supplementary file 11 — Table S3. Distribution of methylation status by recurrence events in TCGA cohort. (DOCX 17 kb) [file 13148_2018_485_MOESM11_ESM.docx]

Additional file 11: Table S3. Distribution of methylation status by recurrence events in TCGA cohort

| Gene | Methylation status | Hypopharyngeal cancer | | | Laryngeal cancer | | | Oropharyngeal cancer | | | Oral cavity cancer | | |
| --- | --- | --- | --- | --- | --- | --- | --- | --- | --- | --- | --- | --- | --- |
|  |  | Recurrence events | | | Recurrence events | | | Recurrence events | | | Recurrence events | | |
|  |  | positive | negative | P† | positive | negative | P† | positive | negative | P† | positive | negative | P† |
| SST | Yes | 3 | 2 |  | 11 | 25 |  | 7 | 33 |  | 51 | 101 |  |
|  | No | 1 | 4 | 0.519 | 19 | 52 | 1 | 2 | 6 | 1 | 42 | 107 | 0.322 |
| TAC1 | Yes | 2 | 2 |  | 13 | 42 |  | 7 | 30 |  | 46 | 91 |  |
|  | No | 2 | 4 | 0.895 | 17 | 35 | 0.390 | 2 | 9 | 1 | 47 | 117 | 1 |
| HCRT | Yes | 3 | 3 |  | 19 | 25 |  | 4 | 27 |  | 46 | 106 |  |
|  | No | 1 | 3 | 0.895 | 11 | 52 | 0.005* | 5 | 12 | 0.310 | 47 | 102 | 0.901 |
| NPY | Yes | 2 | 2 |  | 10 | 28 |  | 8 | 23 |  | 49 | 111 |  |
|  | No | 2 | 4 | 0.895 | 20 | 49 | 0.825 | 1 | 16 | 0.192 | 44 | 97 | 1 |
| GAL | Yes | 3 | 2 |  | 21 | 35 |  | 2 | 15 |  | 48 | 107 |  |
|  | No | 1 | 4 | 0.519 | 9 | 42 | 0.031* | 7 | 24 | 0.595 | 45 | 101 | 1 |

† Chi-squared test, * P<0.05
